# Phocine Distemper Virus: Current Knowledge and Future Directions

**DOI:** 10.3390/v6125093

**Published:** 2014-12-22

**Authors:** Pádraig J. Duignan, Marie-Françoise Van Bressem, Jason D. Baker, Michelle Barbieri, Kathleen M. Colegrove, Sylvain De Guise, Rik L. de Swart, Giovanni Di Guardo, Andrew Dobson, W. Paul Duprex, Greg Early, Deborah Fauquier, Tracey Goldstein, Simon J. Goodman, Bryan Grenfell, Kátia R. Groch, Frances Gulland, Ailsa Hall, Brenda A. Jensen, Karina Lamy, Keith Matassa, Sandro Mazzariol, Sinead E. Morris, Ole Nielsen, David Rotstein, Teresa K. Rowles, Jeremy T. Saliki, Ursula Siebert, Thomas Waltzek, James F.X. Wellehan

**Affiliations:** 1 Department of Ecosystem and Public Health, University of Calgary, Calgary, AB T2N 4Z6, Canada; E-Mails: ppjduign@ucalgary.ca (P.D.); klamy@gmail.com (K.L.); 2 Cetacean Conservation Medicine Group (CMED), Peruvian Centre for Cetacean Research (CEPEC), Pucusana, Lima 20, Peru; E-Mail: mfb.cmed@gmail.com; 3 Pacific Islands Fisheries Science Center, National Marine Fisheries Service, NOAA, 1845 WASP Blvd., Building 176, Honolulu, Hawaii 96818, USA; E-Mails: Jason.Baker@noaa.gov (J.D.B.); michelle.barbieri@noaa.gov (M.B.); 4 The Marine Mammal Centre, Sausalito, CA 94965, USA; E-Mail: gullandf@tmmc.org; 5 Zoological Pathology Program, College of Veterinary Medicine, University of Illinois Urbana-Champaign, Maywood, IL 60153, USA; E-Mail: katie.colegrove@gmail.com; 6 Department of Pathobiology and Veterinary Science, and Connecticut Sea Grant College Program, University of Connecticut, Storrs, CT 06269, USA; E-Mail: sylvain.deguise@uconn.edu; 7 Department of Viroscience, Erasmus MC, 3015 CN Rotterdam, The Netherlands; E-Mail: r.deswart@erasmusmc.nl; 8 Faculty of Veterinary Medicine, University of Teramo, 64100 Teramo, Italy; E-Mail: gdiguardo@unite.it; 9 Department of Ecology and Evolutionary Biology, Princeton University, Princeton, NJ 08544-2016, USA; E-Mails: dobson@princeton.edu (A.D.); grenfell@princeton.edu (B.G.); semorris@princeton.edu (S.E.M.); 10 Department of Microbiology, Boston University School of Medicine, Boston University, 620 Albany Street, Boston, MA 02118, USA; E-Mail: pduprex@bu.edu; 11 Greg Early, Integrated Statistics, 87 Water St, Woods Hole, MA 02543, USA; E-Mail: greg.early@yahoo.com; 12 National Marine Fisheries Service/National Oceanographic and Atmospheric Administration, Marine Mammal Health and Stranding Response Program, Silver Spring, MD 20910, USA; E-Mails: deborah.fauquier@noaa.gov (D.F.); teri.rowles@noaa.gov (T.K.R.); 13 One Health Institute, School of Veterinary Medicine, University of California, Davis, CA 95616, USA; E-Mail: tgoldstein@ucdavis.edu; 14 School of Biology, University of Leeds, Leeds LS2 9JT, UK; E-Mail: s.j.goodman@leeds.ac.uk; 15 Fogarty International Center, National Institutes of Health, Bethesda, MD 20892-2220, USA; 16 Department of Pathology, School of Veterinary Medicine and Animal Science, University of São Paulo, São Paulo 05508-270, Brazil; E-Mail: katia.groch@gmail.com; 17 Marine Mammal Commission, 4340 East-West Highway, Bethesda, MD 20814, USA; 18 Sea Mammal Research Unit, Scottish Oceans Institute, University of St. Andrews, St. Andrews, Fife KY16 8LB, UK; E-Mail: ajh7@st-andrews.ac.uk; 19 Department of Natural Sciences, Hawai’i Pacific University, Kaneohe, HI 96744, USA; E-Mail: bjensen@hpu.edu; 20 Keith Matassa, Pacific Marine Mammal Center, 20612 Laguna Canyon Road, Laguna Beach, CA 92651, USA; E-Mail: kmatassa@pacificmmc.org; 21 Department of Comparative Biomedicine and Food Science, University of Padua, 35020 Legnaro Padua, Italy; E-Mail: sandro.mazzariol@unipd.it; 22 Department of Fisheries and Oceans Canada, Central and Arctic Region, 501 University Crescent, Winnipeg, MB R3T 2N6, Canada; E-Mail: ole.nielsen@dfo-mpo.gc.ca; 23 David Rotstein, Marine Mammal Pathology Services, 19117 Bloomfield Road, Olney, MD 20832, USA; E-Mail: drdrot@gmail.com; 24 Athens Veterinary Diagnostic Laboratory, College of Veterinary Medicine, University of Georgia, GA 30602, USA; E-Mail: jsaliki@uga.edu; 25 Institute for Terrestrial and Aquatic Wildlife Research, University of Veterinary Medicine Hannover 30173, Germany; E-Mail: ursula.siebert@tiho-hannover.de; 26 Department of Infectious Diseases and Pathology, College of Veterinary Medicine, University of Florida, FL 32611, USA; E-Mail: tomwaltzek@gmail.com; 27 Department of Small Animal Clinical Sciences, College of Veterinary Medicine, University of Florida, FL 32610, USA; E-Mail: wellehanj@ufl.edu

**Keywords:** Morbillivirus, pinnipeds, sea otter, CD150/SLAM, phylogeny, pathology, epidemiology, immunity, vaccine

## Abstract

Phocine distemper virus (PDV) was first recognized in 1988 following a massive epidemic in harbor and grey seals in north-western Europe. Since then, the epidemiology of infection in North Atlantic and Arctic pinnipeds has been investigated. In the western North Atlantic endemic infection in harp and grey seals predates the European epidemic, with relatively small, localized mortality events occurring primarily in harbor seals. By contrast, PDV seems not to have become established in European harbor seals following the 1988 epidemic and a second event of similar magnitude and extent occurred in 2002. PDV is a distinct species within the *Morbillivirus* genus with minor sequence variation between outbreaks over time. There is now mounting evidence of PDV-like viruses in the North Pacific/Western Arctic with serological and molecular evidence of infection in pinnipeds and sea otters. However, despite the absence of associated mortality in the region, there is concern that the virus may infect the large Pacific harbor seal and northern elephant seal populations or the endangered Hawaiian monk seals. Here, we review the current state of knowledge on PDV with particular focus on developments in diagnostics, pathogenesis, immune response, vaccine development, phylogenetics and modeling over the past 20 years.

## 1. Introduction

Beginning in late 1987 seemingly unprecedented epidemics spread through pinniped populations from Siberia to Western Europe. The series began with the mass mortality of Baikal seals (*Pusa sibirica*) in land-locked Lake Baikal [1]. The event followed an outbreak of canine distemper virus (CDV) infection in terrestrial mammals and a variety of diagnostic tests later confirmed that CDV was implicated in the seal deaths [1,2]. Shortly afterwards, beginning in April 1988, an epidemic swept through breeding colonies of European harbor seals (*Phoca vitulina vitulina*) around the coasts of the North, Baltic and Irish seas killing up to 18,000 of this species and possibly a few hundred sympatric grey seals, *Halichoerus grypus* [3]. However, while the clinical presentation and pathology were similar to CDV infection, antigenic characterization and gene sequencing demonstrated that the virus was a novel and distinct member of the *Morbillivirus* genus, phocine distemper virus, PDV [4,5,6].

The course and outcome of the 1988 PDV epidemic suggested that this was a “virgin soil” event in previously naïve animals [7] and raised the question of where the virus had originated. Limited serology from archived sera of European seals collected prior to 1988 showed no prior evidence of infection [6,8,9]. An early hypothesis that harp seals (*Pagophilus groenlandicus*) from the eastern Arctic may have been the source of infection was supported by subsequent serological surveys in Norway, Greenland and Canada [10,11,12,13,14].

A symposium and round table discussion was convened in Hannover, Germany, in 1994 to review the current knowledge on marine mammal morbilliviruses [15,16]. Twenty years later, August 2014, a Research and Policy for Infectious Disease Dynamics (RAPIDD) workshop was convened on marine mammal morbilliviruses at Princeton University, USA, to discuss recent advances in research on PDV pathology, pathogenesis, transmission, species susceptibility, immunology and development of vaccination strategies for naïve threatened species such as Mediterranean (*Monachus monachus*) and Hawaiian (*M. schauinslandii*) monk seals, and future directions for research. As an outcome of the workshop and round table discussion, we review the pertinent research in these relevant fields that has been published in the past 20 years and identify knowledge gaps requiring further research investment.

## 2. Antigenic and Molecular Characteristics of PDV

The *Morbillivirus* genus comprises well known pathogens of terrestrial mammals including measles virus (MV) a pathogen of humans and primates, rinderpest virus (RPV) and peste des petits ruminants (PPRV) both pathogens of ungulates and CDV a pathogen of carnivores [17]. A recently identified virus in cats has been proposed as a new morbillivirus of domestic felines, feline morbillivirus (FmoPV) [18]. Although this virus has the same gene organization as morbilliviruses, its pathological and molecular biological features are quite different from those of the conventional morbilliviruses. Among marine mammals, cetaceans (whales, dolphins and porpoises) may be infected by the recently recognized cetacean morbillivirus (CeMV) in which a number of strains are now recognized globally including dolphin morbillivirus (DMV), porpoise morbillivirus (PMV) and pilot whale morbillivirus (PWMV) [19,20,21,22,23,24,25,26,27]. Although not a subject of this review, CDV from terrestrial hosts has caused epidemics among land-locked Baikal seals and Caspian seals (*P. caspica*) since the late 1980s [1,28]. The morbillivirus genome is comprised of a non-segmented, negative-sense, single stranded RNA that varies from 15,500 to 16,050 nucleotides in length and contains six transcription units that encode six structural proteins: nucleocapsid (N), phosphoprotein (P), matrix (M) protein, fusion (F) glycoprotein, hemagglutinin (H) glycoprotein, and the major component of the RNA-dependent RNA polymerase, the large (L) protein. Two non-structural proteins (C and V) sometimes termed virulence factors that interfere with the innate immune response and affect infectivity, are also expressed in infected cells. Preliminary genetic characterization of PDV isolates from the 1988 epidemic using cDNA probes confirmed its membership in the *Morbillivirus* genus as a novel species [5,29,30,31]. Phylogenies of morbilli and paramyxoviruses based on partial [32,33,34,35], and more recently, complete gene sequences [36] consistently place all PDV strains as a monophyletic sister clade to CDV across all genes. Recent evidence points to bats as a potential source of significant paramyxovirus diversity, with some taxa clustering with the CDV/PDV group [37]. Host jumping appears to be relatively common in the *Paramyxovirinae*, and morbilli-like viruses are found in diverse mammals [38]. Final confirmation of the unique identity of PDV was achieved with full genome sequencing using a 1988 isolate in Vero cells expressing the canine receptor CD150 [39]. The genome was 15,696 nucleotides in length with the typical six non-overlapping morbillivirus genes in the order N-P/V/C-M-F-H-L [39]. Like all other morbilliviruses it obeys the rule of six [40].

A second and equally devastating epidemic occurred among European harbor seals in 2002, with a similar temporal and geographic range to the 1988 event [41]. The similarities raised the possibility that the virus had persisted in the region in either marine or terrestrial hosts. Epitope mapping of five structural proteins from the 1988 and 2002 isolates using a panel of monoclonal antibodies found no differences between the PDV isolates, suggesting a high degree of antigenic conservation over the 14 year time span [42]. However, phylogenetic analysis of the wild-type H genes from PDV 1988 and 2002 showed distinct differences between isolates from each epidemic, suggesting that the virus circulating in 2002 had been reintroduced into the North Sea and not maintained in either marine or terrestrial hosts over the intervening interval [43]. Equally intriguing was the discovery that a PDV isolate from a harbor seal stranded in Maine, USA in 2006 during an unusual mortality event was more similar to the Netherlands 1988 isolate than to the European 2002 isolates when compared using the H gene and deduced amino acid sequences [44]. The authors concluded that multiple lineages of PDV may be circulating in endemically infected seal populations in the eastern North Atlantic, a situation analogous to that of CDV in terrestrial carnivores [45]. Given the lifelong immunity and significant cross-protection seen between different morbillivirus strains, the maintenance of multiple strains in seal populations is surprising and further study into survival of strains between epizootics is merited [39].

**Figure 1:**
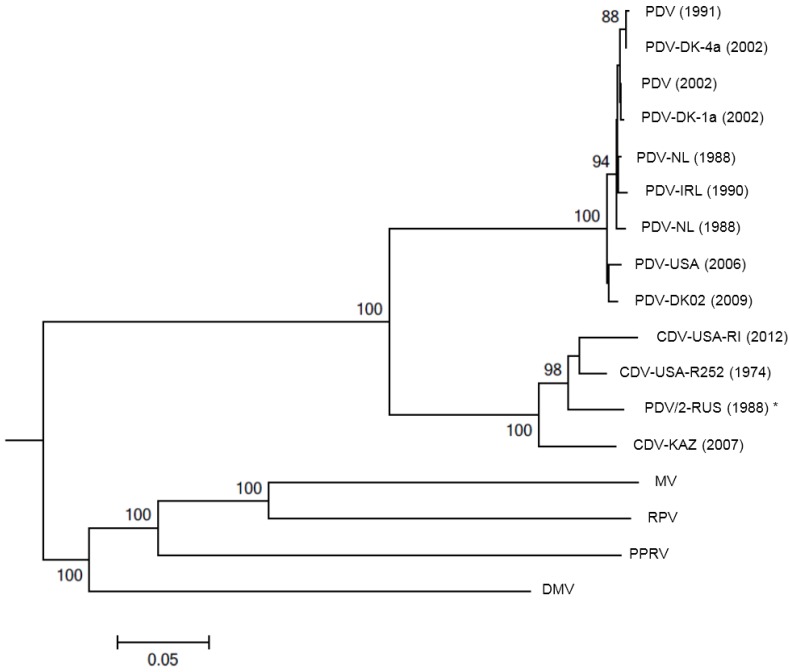
Evolutionary relationships of phocine distemper virus (PDV) isolates to the other morbilliviruses. The neighbor-joining method [46] was used to generate a phylogenetic tree based on the hemagglutinin (H) glycoprotein sequences of seventeen selected PDV, CDV, MV, RPV, PPRV and DMV strains (branch length 1.59). Positions containing gaps were eliminated. Bootstrap analysis [47] was used to indicate the percentage number of trees from 1000 replicates in which the virus H glycoprotein sequences clustered equivalently. Branch lengths are identical to the evolutionary distances, determined using the p-distance method [48], used to infer the phylogenetic tree. Sequence alignments and phylogenetic analysis was performed using MEGA5 [49]. PDV accession numbers (Z36979.1; AF479276.1; AF479277.1; AF479274.1; D10371.1; KC802221; AJ224707.1; HQ007902.1 and FJ648456.1), PDV/2-RUS (1988) * (X84998.1) is generally accepted to be a CDV isolate although the name has not been changed. CDV accession numbers (HM046486.1, USA-RI and USA-R252, in submission). Other morbilliviruses, accession numbers, MV (HM439386.1), RPV (NC006296.2), PPRV (FJ750563.1) and DMV (FJ648457.1), were included to show the evolutionary distances across the genus. Feline morbillivirus was not included in this analysis as it is questionable if this virus is a true morbillivirus.

A phylogenetic tree was constructed *de novo* from published and unpublished H gene sequences of PDV (Europe 1988, 2002, USA 2006), CDV (R252 canine strain, a wild-type raccoon strain, Baikal seal CDV and Caspian seal CDV), RPV, MV and DMV (representing the cetacean morbilliviruses) to demonstrate the evolutionary relationships of PDV isolates within the wider genus (Figure 1). Neighbor-joining methodology was used and the H glycoprotein was chosen as this is under immunological pressure. Furthermore, it is known that there is good correlation between trees generated using H gene sequences and those using the highly variable cytoplasmic tail of the N protein, which is commonly used for morbillivirus genotyping [50]. The phylogenetic tree demonstrates that PDV and CDV cluster into distinct, but evolutionary similar, lineages and each lineage has several variants circulating as wild-type virus with slight variations between PDV isolates from the northeastern and northwestern Atlantic.

## 3. Clinical Signs, Pathogenesis and Pathology

### 3.1. Clinical Signs of Infection

The clinical course of acute PDV infection in free-ranging pinnipeds has most frequently been observed in harbor seals, and occasionally grey, harp and hooded seals, during epidemics or mortality events in Europe and North America [51,52,53,54,55,56,57]. Signs may include pyrexia, serous or mucopurulent ocular and nasal discharges consistent with conjunctivitis, keratitis, ophthalmitis and rhinitis. Respiratory signs include coughing, mucosal cyanosis, dyspnea with interstitial and subcutaneous emphysema in severe cases increasing buoyancy and impeding normal swimming and diving. Females infected during pregnancy are prone to abortion. Moribund seals remain ashore for longer and may develop pressure necrosis and higher than expected ectoparasite burdens. Neurological signs manifest as depression, lethargy, head tremors, convulsions and seizures. Clinical infection has been described in one juvenile harp seal from eastern Canada that was moribund for one week with lethargy, severe conjunctivitis, multifocal epidermal ulceration, mucosal cyanosis and increased lung density on radiographs [53].

### 3.2. Pathogenesis, Cell Receptors and Tissue Tropism

The morbillivirus H glycoprotein is required for attachment to a specific host cell membrane receptor while the F glycoprotein interacts specifically with H to facilitate viral envelope fusion with the host cell membrane [58]. The specific interaction of the viral H and F glycoproteins with the host cell receptor(s) determines host susceptibility, tissue tropism and viral pathogenesis. However, because morbilliviruses infect a number of different cell types *in vivo* including leucocytes, epithelial, endothelial and neural cells, more than one receptor type is required [59].

The complement binding glycoprotein CD46 was first shown to be a competent receptor for MV, possibly with the involvement of a cytoskeletal protein, moesin [58,60]. A related glycoprotein CD150 (or SLAM/F1, signaling lymphocyte activation molecule F1) was demonstrated using *in vitro* techniques to be a principal cellular receptor for MV, RPV and CDV in people, cattle and dogs respectively [61,62]. Recently, expression of CD150 was confirmed on lymphocytes of a wide range of species in the suborders *Caniformia* and *Feliformia* including spotted seals (*Phoca largha*) and walrus (*Odobenus rosmarus*), while both CD150 and CD46 are expressed on harbor seal lymphocytes [63,64,65]. CD150 is a member of the C2 subset of the immunoglobulin superfamily and is expressed on activated B and T cells, constitutively on immature thymocytes, memory T cells, a proportion of B cells as well as activated monocytes and mature dendritic cells [66,67,68]. Phylogenetic research suggests that host CD150 and viral H glycoprotein have co-evolved [69]. Further work confirmed that CD150, but not CD46, is used by wild-type (wt) PDV as a host cell receptor [59]. Use of transfected Vero cell lines containing canine CD150 receptors allow for the efficient isolation of CDV, PDV and CeMV [44,70]. As there is a broad recognition of canine CD150 by a number of morbilliviruses it is hypothesized that cross-species infections are likely to occur in the future [71].

In experimental studies using the ferret-CDV model, it was shown that the H protein is a key determinant of virus interaction with CD150, and that variations in this protein may influence tissue tropism [70,72,73]. Molecular evolution studies of CDV isolates from non-canine carnivores indicate that variation at a small number of key H protein residues involved in binding to CD150 may drive CDV adaptation to new hosts [74]. Although the H-protein sequence is overall not well conserved, two clusters of H amino acid residues involved in CD150 attachment (positions 526–529 and 547–548 and amino acid 552) are highly conserved and possibly facilitate overlapping host ranges between PDV and CDV [43,63,73].

A second mammalian cell surface receptor expressed mainly on the basal-lateral surfaces of epithelial cells, the poliovirus receptor-like protein 4 (PVRL4), also known as nectin 4, has been identified as a receptor for MV, CDV and PPRV [75,76,77,78]. This receptor is also up-regulated by MV in human brain endothelial cells [79]. Recently, PVRL4 was shown to be a receptor for wtPDV, potentially increasing the host range for the virus as it is a commonly expressed surface molecule on mammalian epithelial cells [59]. However, because of the ability of wtPDV to replicate in Vero cells without prior adaptation, it has been proposed that wtPDV may use other host cell molecules such as Toll-like receptors, interferon gamma (IFNγ), interleukin-4 (IL4), IL8, IL10, pro-HB-EGF and the vitamin A receptor (RARa) [59,65]. It has also been postulated that alternate mechanisms of infection must be used by morbilliviruses to enable infection of neural cells that do not express CD150 or PVRL4, and for intra-cerebral spread in subacute-sclerosing panencephalitis, SSPE, in people [80].

Thus, the molecular biology and receptor usage of PDV would presumably parallel that of CDV and MV [77,81]. In laboratory studies, ferrets infected with CDV and macaques infected with MV show initial (5 to 6 days post infection) primary replication in lymph nodes and secondary lymphatic organs including those of the lungs, bronchi, and trachea [82,83,84,85]. Later (12 days post infection), most infected cells of the trachea are of lymphoid or myeloid origin and are located beneath the epithelium. CDV uses CD150 as a receptor to infect lymphocytes and dendritic cells and presumably uses PVRL4 to infect epithelial cells in the respiratory tract and elsewhere in the body where this receptor is expressed on epithelia. The logistical difficulty of working with pinniped species when experimental infections are impossible, may require the use of *in vitro* models. These could be used to identify differences in virus attachment to peripheral blood lymphocytes from harbor, grey and harp seals [86].

### 3.3. Gross Pathology

Based on experimental infectivity studies, the pathogenesis and pathology of PDV infection in harbor seals is similar to that of CDV in dogs [87,88]. Initial viral replication appears to occur in lymphoid cells with secondary dissemination to epithelial and endothelial cells in various organ systems and to the central nervous system [83,87,89]. Histopathology and the immunohistochemical localization of morbillivirus antigen, such as N protein, in tissues of naturally-infected harbor, grey and harp seals have been described in detail and extensively reviewed [53,90,91,92,93,94]. In summary, pneumonia is the principal gross lesion with variable consolidation, atelectasis, congestion, edema and emphysema. The latter is rarely limited to the lungs with combinations of interlobular, sub-pleural, mediastinal, pericardial, retroperitoneal and subcutaneous emphysema. Bronchial and mediastinal lymphadenopathy are frequent. Primary PDV pneumonia is often complicated by concurrent parasitic pneumonia (*Parafilaroides spp*.), bacterial infections (*Bordetella bronchiseptica, Streptococcus spp. Clostridium spp*. among others) and viral co-infections (*Phocid herpesvirus 1*, *Influenza A virus*). Consequently there may be suppurative and hemorrhagic pneumonia, mucopurulent exudates in airways, pleuritis, hematomas and infarction. Dermatitis is often manifested as focal or locally extensive areas of hyperkertosis on ears, eyelids and foot pads of terrestrial carnivores infected by CDV [95]. Similar pathology has not been reported for harbor seals, the pinniped most susceptible to PDV. However, focal crusting on the dorsal surface of the flippers, head, trunk and tail was described for a juvenile harp seal and hooded seal (*Cystophora cristata*) stranded on the US Atlantic coast [55].

### 3.4. Histopathology

Characteristic histopathologic lesions of PDV may be observed in the respiratory tract, lymphoid system, various epithelia and the central nervous system [51,53,54,90,91,94,96]. Bronchointerstitial pneumonia is characterized, in cases uncomplicated by secondary infections, by serofibrinous alveolar exudates containing leukocytes and macrophages. Serofibrinous exudate is replaced in subacute cases by hyaline membranes and in more chronic cases, fibroplasia in alveolar septa and infiltration by lymphocytes, plasma cells and other leukocytes. Initial viral replication in type I pneumocytes during acute infection results in their necrosis and eventual replacement by type II pneumocytes in more sub-acute cases. Syncytia are found in bronchioles, alveoli and peri-bronchiolar glands but these are generally less numerous and smaller than the equivalent in cetaceans infected by CeMV [93]. Acidophilic intracytoplasmic inclusion bodies (ICIB) and intranuclear inclusion bodies (INIB) of respiratory epithelium have been observed throughout the tract including bronchiolar gland epithelium, type II pneumocytes and in syncytia. Inclusions are generally discrete, ovoid (10–20 µm) with distinct borders. Similar inclusions may be present in transitional epithelium of the renal pelvis and urinary bladder, biliary and pancreatic ducts, tonsils and gastro-intestinal epithelia, and conjunctiva.

**Figure 2:**
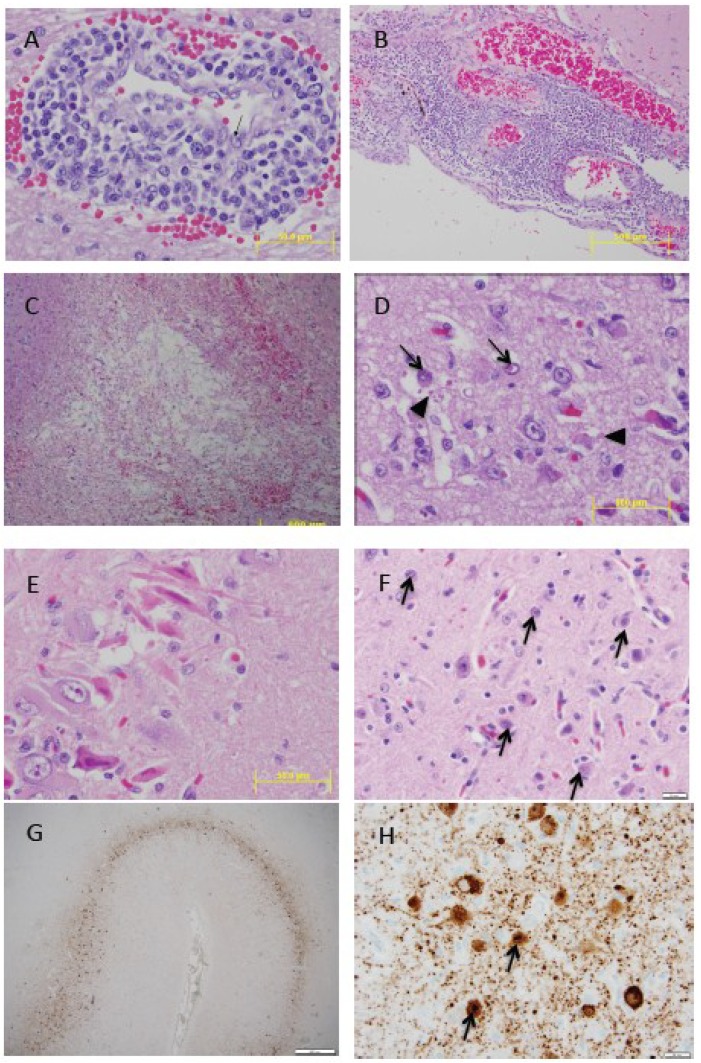
(**A**) Grey seal (*Halichoerus grypus*) cerebrum showing expanded Virchow Robin space containing lymphocytes. Activated endothelial cell (arrow). H&E stain (**B**) Grey seal cerebrum with marked non-suppurative meningoencephalitis. H&E stain (**C**) Grey seal cerebral white matter with focal malacia centrally and peripheral focal hemorrhage. H&E stain (**D**) Grey seal cerebrum with acidophilic intranuclear inclusion bodies (INIB, arrow) and intracytoplasmic inclusion bodies (ICIB, arrow heads). H&E stain (**E**) Grey seal hippocampus with acute focal neuronal necrosis. H&E stain (**F**) Harp seal (*Pagophilus groenlandicus*) cerebrum with numerous INIBs (arrows). H&E stain; (**G**) Harp seal cerebrum showing laminar distribution of morbillivirus antigen. Immunohistochemistry (IHC) using primary antibody against the nucleocapsid (N) of canine distemper virus (VMRD, Inc., Pullman, WA, USA); (**H**) Harp seal cerebrum. Neurons with strong staining of morbillivirus antigen in cytoplasm and nuclei (arrows). IHC stain as described. The grey seal tissues are from a pup that stranded in February 2006 in Maine, USA, with neurological clinical signs [56]. The harp seal tissue is from a juvenile that stranded on Prince Edward Island, Canada, in May 1991 with similar clinical signs [53].

Marked necrosis and depletion of lymphocytes in spleen, thymus, gut-associated lymphoid tissue and peripheral lymph nodes is characteristic. Acidophilic ICIBs may be observed in lymphoid tissues and severely depleted follicles may have central syncytia. Non-suppurative encephalitis in harbor and harp seals is remarkably similar to those of spontaneous CDV in dogs [97,98]. The distribution is generally cerebral and often with a laminar or multifocal pattern of neuronal and glial necrosis in the cerebral cortex, mononuclear perivascular cuffing, astrocytosis, microgliosis, neuronophagia and focal demyelination in cerebral white matter. Acidophilic ICIBs and INIBs are frequently seen in neurons and astrocytes (Figure 2). In dogs, an acute and chronic or relapsing manifestation of demyelinating CDV infection has been described [98]. The acute phase occurs around three weeks post infection and coincides with the period of viral-induced immunosuppression [99]. These animals may progress to death, recover after mild or subclinical illness, or develop a chronic or relapsing disease with persistence of the virus in the brain and progression of demyelination mediated by immunopathological mechanisms [98,100]. While it is plausible that this may also occur in pinnipeds with PDV infection, it has not been demonstrated. Neither is there evidence for an old dog encephalitis (ODE)-like syndrome in pinnipeds. ODE is an extremely rare condition in dogs manifesting as neurological deficits years after primary CDV infection. It is characterized by an intense inflammatory reaction in the forebrain (frontal cortex, basal nucleus, pyriform lobe, rostral thalamus, and rostral subcortical white matter) with sparing of the hindbrain (cerebellum and caudal brain stem) and occipital cortex (grey and white matter). Histologically, there are prominent perivascular cuffs, neuronal necrosis, astrocytosis, formation of multinucleated giant cells and focal demyelination with ICIBs in astrocytes and giant cells [101].

PDV–associated dermatitis has been documented in a harp and hooded seal characterized by a focally thickened epidermis with three distinct layers: a deeply eosinophilic superficial layer of orthokeratotic hyperkeratosis, a zone of hyperplastic basal cells, and middle band with numerous necrotic syncytial cells some of which with as many as 30 to 40 nuclei. These areas resembled syncytial zones rather than discrete syncytial cells. Hair follicle infundibula had similar changes. Eosinophilic ICIBs were observed in epidermal, follicular and sebaceous cells but INIBs were rare. Syncytia were also present in sebaceous glands [55].

### 3.5. Age-Specific Pathology

The high mortality resulting from the 2002 PDV epidemic in Europe enabled a detailed pathological assessment of 369 harbor seal stranded on the coasts of The Netherlands [94]. The seals were aged based on dentin layers in teeth and classed as <1, 1 to 2 years, and 3 years or older. Analysis of the pathology and ancillary diagnostic results enabled insights into the distribution of lesions, temporal presence of viral antigen or genome in organs, and influence of co-infections. The data confirmed the differential temporal occurrence of PDV in lung and brain. As in dogs with CDV, PDV virus persisted much longer in the brain than in lung. As in the 1988 epidemic, a greater proportion of older animals had PDV neutralizing antibodies. This study supported the hypothesis that there was greater mortality among younger age classes and age-related immune compromise in younger seals. The course of disease in older seals may also be longer enabling the development of an immune response. A second age-related observation was the development and severity of extra-thoracic emphysema that increased with age and was possibly related to the more chronic course of disease in older animals or their diving behavior. The study further enabled documentation of the role of PDV-induced immunosuppression in the promotion of secondary bacterial and parasitic infections in older animals.

## 4. Diagnosis

The clinical signs of respiratory tract infection seen in seals with PDV and the associated oculo-nasal discharge and conjunctivitis are nonspecific and may be seen with *Influenza A virus* and other infections [102,103]. Similarly, the neurological impairment is similar to that seen following ingestion of marine algal biotoxins such as domoic acid [104]. Gross pathology in cases of acute respiratory infection is highly suggestive of PDV but not pathognomonic. Serology is most useful for population surveillance and less so as a diagnostic tool for individual animals. Antigen-capture enzyme-linked immunosorbent assays (ELISA) have been used on tissue homogenates [105]. Histopathology, immunohistochemistry and molecular diagnostic techniques as described below are currently the most commonly used laboratory tests for confirmation of infection.

### 4.1. Serology

Because serological tests do not always measure the same analyte (e.g., IgM *versus* IgG, antibodies directed against different viral antigens), results from different tests are not always comparable. Virus neutralization (VN), and plaque reduction (PR) tests are the most extensively used to determine the presence of antibodies against morbilliviruses in the blood of a large number of pinniped species [6,10,12,106,107,108]. These are also the only available serological tests that can different between CDV and PDV infections in which a differential antibody titer of 4-fold or greater indicates homologous as opposed to heterologous exposure. While both tests are capable of detecting circulating antibodies, PR testing has the added advantage in that it can be used on the more degraded or hemolysed and whole blood samples usually obtained from beached carcasses or from animals harvested for food by indigenous hunters. Testing is usually done for both assays using standard Vero cells. However, use of Vero.DogSLAMtag cells, expressing canine CD150 receptor, which are more sensitive to virus growth, permits reduction of testing time by 1–2 days for both the VN and PRN tests, [109], Saliki, unpublished observations]. CDV, PDV and CeMV all share numerous antigenic determinants that are recognized by infected hosts. It has been shown that serum antibodies raised against one morbillivirus will neutralize the homologous virus to a higher titer than the heterologous morbilliviruses [12,108,110]. This allows the determination of the species of morbillivirus responsible for infection and greatly increases our knowledge regarding the epidemiology and transmission of specific viruses in free-living populations worldwide [12,106,111].

### 4.2. Histopathology and Immunohistochemistry

The identification of acidophilic or amphophilic ICIBs or INIBs in hematoxylin and eosin (H&E) stained tissue sections is strongly suggestive of morbillivirus infection. When dealing with potentially new host species, or new areas of disease emergence, it is recommended to use ancillary tests such as immunohistochemistry to confirm the presence of morbilliviral antigen [51,53,54,97]. Immunohistochemistry is also extremely useful if the classic histological lesions or inclusions are no longer visible or equivocal as can occur in more chronic cases and in animals where the viral lesions are masked by severe inflammation or necrosis caused by secondary infections. Currently, most laboratories use an avidin-biotin complex (ABC) technique and either a primary monoclonal antibody against the nucleocapsid (N) of CDV (VMRD, Inc., Pullman, WA, USA) or polyclonal rabbit anti-CDV nucleoprotein [112,113].

### 4.3. Reverse Transcription Polymerase Chain Reaction (RT-PCR)

By far the most common PCR assay used for detection of PDV is the “universal” morbillivirus primer set that amplifies a 486 bp fragment of the P gene, followed by nested PCR using specific primer sets that amplify a 384 bp fragment of the PDV and CDV P genes respectively, to ascertain which virus is present [114,115]. This assay has been used to detect PDV in live animals (whole blood, nasal, pharyngeal or ocular swabs) and in tissues such as trachea-bronchial lymph nodes, spleen, liver, kidney, lung and brain from carcasses. This assay was used to test tissue samples from the 2002 European harbor seal epidemic [116] and more recently from nasal swabs and archived tissues from Northern sea otters (*Enhydra lutris kenyoni*) in Alaska [117]. Stanton *et al.* [113] modified the P gene primers to shorten the amplicon to a 149 bp fragment for use in formalin-fixed paraffin-embedded tissues. The assay maintained the sensitivity to differentiate CDV and PDV, and was useful for determining that CDV was present in formalin-fixed paraffin-embedded tissues from a Caspian seal; and PDV in harp, hooded and European harbor seal tissues. A quantitative real-time PCR assay was used during the North Sea European harbor seal epidemic in 2002 to test samples from sympatric grey seals for evidence of PDV infection shortly after the peak of the epidemic [118]. The TaqMan probe and primers were designed using the PDV H gene sequence from the 1988 epidemic (GenBank accession no. D10371). Primers were also designed to amplify a fragment of the grey seal b-actin gene, as a housekeeping gene to determine the integrity of the cDNA and to confirm PCR efficiency of the assay. Incorporation of the b-actin assay is useful for better interpretation of negative results when using any of the morbillivirus assays. Real-time PCR was chosen over conventional RT-PCR as it allowed for both detection and quantification of the number virus copies in the sample. More recently, Earle *et al.* [44] designed additional PDV primers to amplify the H, F, M, and P genes using previously published PDV sequences, for confirmation of the USA 2006 PDV outbreak in harbor seals. Selected fragments of the PCR products are then sequenced for virus identification and phylogenetic analysis.

Recently, a one-step duplex quantitative RT-PCR assay (RT-qPCR) based on TaqMan probe technology was developed to quantify PDV along with the glyceraldehyde-3-phosphate-dehydrogenase (GAPDH) gene to simultaneously assess RNA quality [119]. This approach will be useful to reduce the likelihood of false negative diagnosis in degraded field samples. Another recent development is a pan-marine mammal morbillivirus semi-nested RT-PCR using a degenerate set of primers targeting conserved sequences of the P protein has been described [120], which detects both pinniped and cetacean morbilliviruses. Such an assay is useful for detecting morbilliviruses in multiple marine mammal species.

### 4.4. Virus Isolation

Morbilliviruses are notoriously difficult to isolate and propagate in cell culture [121]. PDV was first isolated by experimentally infecting dogs with spleen, lung, and lymph node homogenate from three naturally-infected infected seals [6]. The first direct isolation was from a primary kidney cell culture from a naturally-infected harbor seal [122]. Co-cultivation of buffy coat leukocytes from infected seals with Vero cells has also been successful [123]. More recently, Vero cells expressing the dog CD150 receptor were tested in order to develop a cell culture and isolation methodology that may be more useful for isolating and better understanding the phylogeny and evolution of marine mammal morbilliviruses [109]. Result showed that PDV replication in Vero.DogSLAMtag cells progressed rapidly (on the order of days) and required fewer passages, whereas virus replication in primary seal kidney and Vero cells took much longer (on the order of weeks) and required multiple passages [109]. Thus, these VeroSLAM cells are now used routinely for isolation of marine mammal morbilliviruses, including PDV.

## 5. Immunology, Species Susceptibility and Vaccination

### 5.1. Immune Response to PDV

Based on natural and experimental infection, harbor seals appear more susceptible to PDV than grey seals [51,124]. Species susceptibility may be due to multiple factors and include host-specific factors such as cell receptors and differences in immune response such as antigen processing and presentation, or cytokine production. Serologic surveys in Europe during and after the first PDV epidemic showed higher seroconversion rates in grey seals [125,126]. Harbor seals that died from PDV infection rarely had antibodies directed against the important F and H glycoproteins but had antibodies directed towards internal N and P glycoproteins of PDV and CDV and in this regard were similar to dogs that developed clinical CDV [30,127,128]. Further studies based on the precipitation of radiolabelled CDV proteins by sera from naturally infected grey and harbor seals from North America confirmed that grey seal sera strongly precipitated N protein and the H and F glycoproteins [129]. By contrast, significantly fewer harbor seal sera precipitated the envelope glycoproteins and responses were weaker than with grey seal serum. Harbor seals that died from PDV encephalitis or pneumonia precipitated the N protein alone or reacted weakly with the envelope glycoproteins [129].

Similar studies on sera from clinically normal seropositive Canadian harp and hooded seals showed that harp seal sera reacted similarly to that of grey seals [12]. Significantly more harp seal sera (18 of 20) precipitated N, F and H glycoproteins that those of hooded seals (7 of 20) while three seropositive ringed seal (*Pusa hispida*) sera precipitated N and F [12]. Thus, species differences in humoral immune response may be an important determinant in PDV susceptibility. 

Differential mortality within the European harbor seal metapopulation during the 1988 and 2002 epidemics prompted studies of genetic population structure, genome wide genetic variation and variation in immune response genes [65,130,131,132]. Using microsatellite loci Goodman [130] demonstrated that despite their potential mobility, harbor seal populations in the European epizootic area showed strong genetic structuring. This would allow for differential distribution between populations of alleles contributing to susceptibility at functional loci, but overall levels of genetic variation were similar in populations experiencing high and low mortality. Recently fine scale population structure was identified over short geographic distances within Scandinavian populations [133]. Further examination of genetic variation using microsatellites and single nucleotide polymorphisms (SNPs) in the Wadden Sea harbor seal population (which showed high PDV mortality in both epidemics) demonstrated a link between individual levels of inbreeding and lungworm infection [132,134]. Whether individual inbreeding is relevant to PDV mortality remains to be tested, but the latter studies do establish a link between individual genetic variation and the response to an infectious disease in harbor seals.

Initial characterization of locus complement and variation of major histocompatibility (MHC) class I genes in eight harbor and four grey seals, found that both species had two expressed MHC class I lineages comprising one classical polymorphic lineage, a second non-classical non-polymorphic class I gene, plus a non-expressed pseudogene [131]. The highly polymorphic lineage showed the typical pattern of diversity for MHC genes consistent with diversifying pathogen driven selection, but higher levels of variation in grey seals (with 12 genes, 26 allotypes, 1–4 allotypes per locus) compared to harbor seals (with 6 genes, 18 allotypes, 1–4 allotypes per locus). It is still to be determined whether variation at MHC loci contributed to intra and interspecies differences in susceptibility to PDV at a population level. Nevertheless, the findings complement the previous studies on humoral immune response suggesting a greater capacity to respond to and neutralize PDV by grey seals than harbor seals [30,129].

Variation at targeted candidate immune genes was assessed in a sample of seals that either died from infection or survived and from several geographic locations [65]. The selected genes included morbillivirus receptors (SLAM and CD46), proteins involved in immune detection (TLR2), immune regulation (IFNG, IL4, IL8, IL10) and proteins involved in disease physiology (RARa, vitamin A receptor). No variation was found across Europe in protein coding domains for SLAM or CD46, but SNPs were found in SLAM intron 2, and exon 1 of IL8 and RARa. The available sample sizes did not have sufficient power to resolve a significant association with disease status, but consistent with the microsatellite data, there was significant differentiation of allele frequencies at the SNP loci among populations. This was a first attempt to investigate the association between genetics and disease susceptibility for wildlife using samples from an actual morbillivirus epidemic. Future studies incorporating full genome sequencing of the host species should enable a more refined analysis of the role of host genetics in the immune response and disease susceptibility [65,135].

The immunosuppressive effects of CDV on canine lymphocytes was demonstrated *in vitro* forty years ago [127]. More recent studies have shown that pro-inflammatory cytokines (IL-1β, IL-6, IL-12 and TNFα) are dominant in early infection in dogs [136,137]. As infection spreads, the response switches to an anti-inflammatory Th2 profile (IL-4, IL-10 and TGFβ) [138,139,140]. An *in vitro* study using mitogen stimulated and un-stimulated harbor seal lymphocytes demonstrated a very similar pattern as seen in dogs with switching to a Th2 response occurring between 24 h and 48 h post infection [141]. Assuming these results reflect natural morbillivirus infection, it is a plausible mechanism for the profound generalized immunosuppression seen in PDV infected seals.

### 5.2. Vaccines and Vaccination Strategies for Free-Living Pinnipeds: Hawaiian Monk Seal Case Study

Vaccination of free living wildlife has rarely been performed to protect the target population from infectious disease, with the notable exception of extensive vaccination of some vectors of zoonoses, such as rabies vaccination of raccoons in the eastern U.S or foxes in Europe. When vaccinating wildlife, vaccines must be selected that can be feasibly given to wildlife effectively, that confer a suitable duration of immunity for the species and disease of concern, and that do not disrupt disease surveillance in the target population. Marine mammals have been vaccinated when in rehabilitation settings to protect them from disease during care or after release [142,143], but vaccination of free living marine mammals is logistically difficult due to their aquatic life. In general, the use of attenuated live morbillivirus vaccines is contraindicated because of the potential risk of disease to the vaccinated animal, and potential for spread to in-contact animals [144,145]. However, during the 1988 PDV epidemic commercially available attenuated CDV vaccine was successfully used to immunize harbor and grey seals and vaccinated pregnant grey seals transferred antibodies to their pups [125,142]. Experimental inactivated and subunit CDV vaccines were also used with mixed success in rehabilitated harbor seals [123,143,146].

To date, vaccination has not been used on free living marine mammals. The prospect of doing so is fraught with challenges not least of which is the administration of a vaccine by injection. If an inactivated vaccine is used, then a second booster shot may be required to achieve an adequate level of protection. Nevertheless, the challenge may be justified when highly endangered species such as the Mediterranean monk seal [147] or the Hawaiian monk seal are considered at risk.

The Hawaiian monk seal is among the rarest of pinnipeds, with approximately 1200 individuals remaining throughout the Hawaiian Archipelago [148]. Currently, infectious disease is not known to be limiting monk seal recovery, and serology and post mortem results indicate that the population is naïve to PDV [149]. However, the species has extremely low genetic diversity [150], and although their susceptibility to morbilliviruses is unknown, DMV and CDV have been reported in cetaceans and domestic dogs in the Hawaiian Islands [25] and vagrant pinnipeds from the eastern North Pacific, where PDV has recently been reported [117], have been observed on Hawaiian beaches.

Considering the devastating effects these viruses can have on phocid populations, the Hawaiian monk seal’s low abundance, low genetic diversity, and the potential for exposure to PDV and other morbilliviruses, planning is underway to prevent or mitigate a potential epidemic using disease surveillance (serology and necropsy) coupled with vaccination.

The vaccination plan incorporates three elements: vaccine selection, captive animal testing for safety and efficacy, and vaccination of free-ranging seals. To date, the first two elements have been completed. The candidate vaccine is recombinant CDV (monovalent recombinant canary pox vector expressing CDV antigens, Purevax, Merial) licensed for use in ferrets in the U.S. and used in zoological collections [151]. It is the only CDV vaccine recommended by the American Association of Zoological Veterinarians (http://www.aazv.org) for use in wild carnivores and it is approved generically for use in Hawaii. Availability of this vaccine is a limitation to its use, as the product has been on manufacturer backorder for two years. Without greater certainty regarding the vaccine's future availability, development and testing of a new vaccine will be required, which may delay implementation of the vaccination plan.

Safety and efficacy trials conducted on captive harbor and Hawaiian monk seals demonstrated no adverse reactions and no shedding of canary pox [152]. All subjects developed positive CDV (though not PDV) titers after receiving a booster approximately one month following initial vaccination. The vaccine has also proven to be a safe and effective prophylactic treatment for captive southern sea otters (*E. lutra nereis*) [153].

The optimal strategy for vaccinating free-ranging Hawaiian monk seals is currently under investigation. Low population abundance, one of the risk factors for an outbreak, also makes vaccinating a significant portion of the population tractable. Monk seals tend to haul out singly or as small groups on the beach, are individually identifiable, and are readily approachable. Thus, vaccine may be administered either by brief capture and physical restraint or using a pole syringe.

It has yet to be resolved whether to: (1) vaccinate only in response to an outbreak; (2) conduct prophylactic vaccination; or (3) combine both approaches. Hawaiian monk seals are distributed in a metapopulation comprising many subpopulations spanning the 2500 km-wide archipelago [154]. Most of the population resides in the remote Northwestern Hawaiian Islands (NWHI), associated with small islands and atolls, all but one of which are reachable only by boat. In contrast, a smaller but growing population occurs in the main Hawaiian Islands (MHI), home also to over one million people [155]. Because of the remote location, monk seals in the NWHI are monitored for at most a few months during summer. As such, detecting an outbreak in the NWHI could be delayed or completely escape detection. Thus, response vaccination is not a viable option. By contrast, monk seals in the MHI are monitored year round by volunteer observers and the general public. An outbreak of disease here would likely be reported so response vaccination may be a viable control option.

Currently, a SEIR (Susceptible, Exposed, Infectious, Recovered) compartmental model is under development to simulate trajectories of morbillivirus outbreaks in MHI seals. Contact rates will be estimated using social network analysis of individual association data, and a plausible range of other key parameters (such as latency, duration of infectious period, *etc.*) will be extrapolated from other species and outbreaks. Efficacy of response vaccination in the face of a morbillivirus outbreak in the MHI will be evaluated. A key question is whether an adequate response can be mounted to immunize a sufficient portion of the population in time to halt transmission. With regard to a prophylaxis, the proportion of each subpopulation that must be vaccinated in order to achieve herd immunity will be estimated, along with the uncertainty surrounding these estimates.

## 6. Epidemiology

### 6.1. Transmission and Persistence

In general morbilliviruses are transmitted horizontally by the respiratory route or by contact with oral, respiratory, and ocular fluids and exudates containing the virus [95,156]. Close contact between affected and susceptible animals is probably required due to the relative fragility of enveloped PDV in the external environment. For the same reason, transmission by fomites is probably not common. The highly aggregated nature of seal haul out sites would certainly predispose them to aerosolized virus from infected conspecifics [157]. Depending on the species, seals aggregate seasonally for breeding and molting which would also favor transmission of a highly infectious respiratory virus. It is noteworthy that epidemics or outbreaks of PDV among harbor seals in both Europe and New England began in spring during the pupping/breeding season or at winter haulout sites [41,44,54,158]. Other modes of horizontal transmission are also possible based on the distribution of PDV antigen in epithelia of the urinary tract and skin but these are probably relatively unimportant [51,53,55,88]. Transplacental transmission of morbilliviruses has been documented in dogs and is likely in cetaceans [159,160,161]. However, while abortion and stillbirth were features of the harbor seal 1988 and 2002 epidemics, vertical transmission has not yet been documented in pinnipeds.

For horizontally-transmitted, highly-immunogenic infectious agents like morbilliviruses, there is generally thought to be a critical community size below which infection cannot persist without continued introduction of individuals with productive viral infection [162]. The number of susceptibles, the latent and infectious periods are also critical factors [163]. For MV, models predicted a threshold of at least 250,000 to 500,000 people in a randomly mixing population [164,165,166] while an age-structured model for harbor seals in western Europe predicted a requirement for a population of 300,000 [167]. However, CDV can persist among low-density populations of terrestrial carnivores that have a patchy distribution, live in small social groups, and tend to be territorial. Under these conditions continued transmission of the virus probably requires large spatial scales or multi-host transmission for persistence as has been described for wolves, coyotes and cougars in the Greater Yellowstone Ecosystem [168]. In this system recurring CDV-associated multi-host mortality events are a feature of the disease ecology [168,169]. A parallel scenario of continued transmission, viral persistence and repeated disease outbreaks appears to be occurring among the phocid seal (harp, hooded, ringed, grey and harbor) of the northwestern Atlantic [170].

### 6.2. Global Distribution of PDV

The emergence of PDV as an agent of mass mortality in 1988 changed our perception of the role of infectious diseases in marine mammal ecology and population dynamics. Indeed, following the first epidemic, Harwood and Hall [157] posited that such stochastic events likely play a more important role in population dynamics than density dependent factors. Certainly the magnitude of PDV on harbor seals in western Europe is reminiscent of the impact that other morbilliviruses, RPV and MV, had on previously naïve populations [171,172,173]. Ascertaining the role of endemic morbillivirus infection in regulating marine mammal populations in the western North Atlantic, and the potential for affecting long-term population persistence, particularly of harbor seals, in the eastern North Atlantic is more problematic. The recent emergence of a PDV-like virus in the North Pacific in previously naïve host species, but without large-scale mortality has further complicated an already complex epidemiology (Table 1).

**Table 1:** Marine Mammals in which PDV has been detected.

Ocean Province	Species	Pathology	Serology	PCR	Reference
**Eastern North Atlantic**	*Phoca vitulina*	Yes	Yes	Yes	[4,5,6,28,29,51,91,92,94,96,174]
(Inc. North, Baltic and Irish Seas)	*Halichoerus grypus*	No	Yes	No	[28,90,92,125,126,142]
**Arctic**	*Cystophora cristata*	No	Yes	No	[11]
(Inc. Greenland, Barents, White and Norwegian Seas)	*Pagophilus groenlandicus*	No	Yes	No	[10,11,14]
	*Pusa hispida*	No	Yes	No	[14]
**Western North Atlantic**	*Phoca vitulina*	Yes	Yes	Yes	[49,106,175]
(Eastern Canadian Arctic to Caribbean)	*Halichoerus grypus*	Yes	Yes	Yes	[13,56,106,175]
	*Pagophilus groenlandicus*	Yes	Yes	Yes	[11,12,53,55]
	*Cystophora cristata*	Yes	Yes	Yes	[11,12,56]
	*Pusa hispida*	No	Yes	No	[11,12]
	*Odobenus rosmarus rosmarus*	No	Yes	No	[107,176]
**Eastern North Pacific and Bering Sea**	*Phoca vitulina richardsii*	No	No	No	[6,106,177,178,179]
	*Pusa hispida*	No	No	No	[6]
	*Phoca largha*	No	No	No	[6]
	*Histriophoca fasciata*	No	No	No	[6]
	*Erignathus barbatus*	No	No	No	[6]
	*Odobenus rosmarus divergens*	No	No	No	[6]
	*Eumetopias jubatus*	No	No	No	[6,180]
	*Callorhinus ursinus*	No	No	No	Gulland unpublished
	*Enhydra lutris kenyoni*	No	Yes	Yes	[117,179,181,182]
	*Enhydra lutris nereis*	No	No	No	[179]
	*Zalophus californianus*	No	No	No	Gulland unpublished
	*Arctocephalus townsendi*	No	No	No	Gulland unpublished
**Western North Pacific**	*Phoca vitulina stejnegeri*	No	Yes	No	[183,184]
(Sea of Okhotsk, Sea of Japan, Yellow Sea)	*Eumetopias jubatus*	No	Yes	No	[184]
	*Phoca largha*	No	Yes	No	[184]
**Southern Oceans**					
New Zealand	*Phocarctos hookeri*	No	Yes	No	[185,186]
	*Arctocephalus forsteri*	No	Yes	No	[185,186]
Australia	*Arctocephalus pusillus doriferus*	No	No *	No	[187]
Antarctica	*Lobodon carcinophagus*	No	No	No	[111,188]#
	*Hydrurga leptonyx*	No	No	No	[188]
	*Leptonychotes weddellii*	No	No	No	[111,188]
	*Ommatophoca rossii*	No	No	No	[111]

* Sera tested against Lederle strain CDV in plaque reduction SNT on VeroDogSLAMtag cells; # CDV serology only.

#### 6.2.1. Western North Atlantic

Retrospective studies indicate that PDV has been circulating among several phocid seal species, the Atlantic walrus and polar bears in the western North Atlantic since at least the 1970s [12,13,106,107,125,158,175,176,189]. The highly gregarious and seasonally aggregated harp seal [190] appears to be a key species in the disease ecology of PDV in this ecosystem, with a population size well above estimates required to maintain endemic infection [167]. The historical, pre-European colonization, estimates suggest that there were at least eleven million animals in Canadian waters with only a moderate decrease to eight million in 2011 [191]. Breeding age females sampled in the Gulf of St. Lawrence between 1988 and 1993 were shown to have 83% PDV seropositivity, consistent with endemic infection [12]. This high level of herd immunity precludes large-scale mortality in a population in which most pups will have maternal antibody and susceptibility limited to a short window when maternal immunity wanes and acquired immunity provides life long protection. In keeping with this, the only confirmed PDV mortality among harp seals has been in juveniles dispersing away from the breeding colonies in spring [53,55].

**Figure 3:**
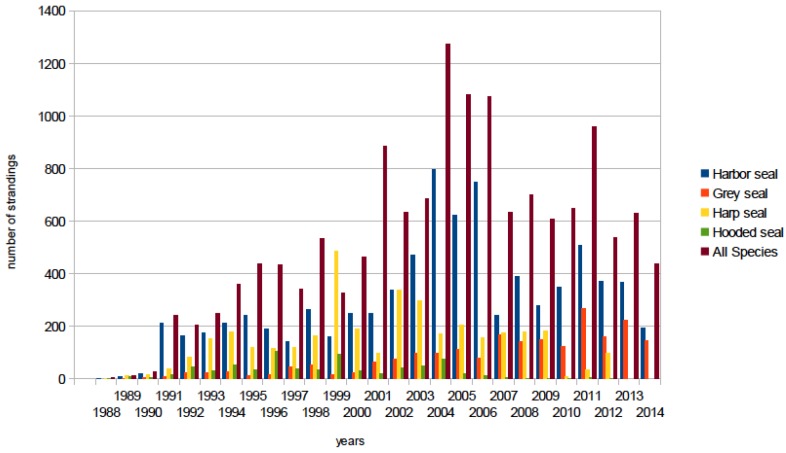
Pinniped strandings by species for the US Atlantic coast for the period 1988 to 2014. The peaks in strandings in 1991/1992; 1998/1999, and from 2004 to the end of 2007 were associated with confirmed PDV infection in harbor [54], harp and hooded seals [55], and grey seals [56].

In the eastern Canadian Arctic it was found that the prevalence of PDV antibody in ringed seals was significantly higher where they were sympatric with migratory harp seals [12]. It is also likely that harp seals act as a reservoir of infection for the less gregarious hooded seals, with whom they share whelping patches on the sea ice in March, and the relatively small Atlantic walrus population, with whom they are also seasonally sympatric [12,107,176]. From late winter though early summer, harp seals are also sympatric with both grey and harbor seals in the Gulf of St. Lawrence, Maritime Canada and along the New England coast. As with harp seals, high levels of seropositivity (73%) were found in adult grey seals sampled in eastern Canada between 1980 and 1994 [106]. Furthermore, as with harp seals, grey seals are a highly gregarious species aggregating in huge numbers for the winter (January-February) breeding season and early spring molt, and wide dispersal of pups of the year [192,193,194,195]. Like the harp seal, the grey seal population in eastern Canadian waters is large and expanding with growth by an estimated 975% between 1977 and 2010 to approximately 348,900 animals [196]. The New England grey seal population has greatly expanded with establishment of breeding colonies in Maine and Massachusetts [193,197]. By contrast, the PDV antibody prevalence in harbor seals in this region over the same period was only 37% [106], but still much higher than in European harbor seals (11%) in post-epidemic years [198]. It was suggested that the smaller population size of this species in eastern Canada and Atlantic USA, its more fragmented distribution, and less gregarious behavior would not be sufficient to maintain endemic infection without contact with either grey or harp seals [106]. Furthermore, the lower level of herd immunity in harbor seals would leave them vulnerable to periodic mortality events. This indeed appears to be the case with a confirmed PDV mortality event occurring along the Atlantic coast from eastern Canada to Long Island, New York, over the winter of 1991/’92 that was preceded by mortality in juvenile harp seals in eastern Canada in spring 1991 [53,54]. Juvenile harp and hooded seals stranded on the US Atlantic coast in increased numbers from 1998 to the end of 1999 (Figure 3). The event was again associated with circulating PDV [55] and may have precipitated the prolonged increase in multi-species mortality through the first decade of the century in the New England region (Figure 3). This latter unusual mortality event included the first cases of clinical disease and death associated with PDV in grey seals in North America [44,56] and the first isolation of a North American strain of PDV from a harbor seal from the US Atlantic coast [44]. The phylogenetic relationship between this isolate, isolates from the two European epidemics, and the harp seal PDV have yet to be established. Preliminary investigations suggest that mutations in the F and M genes of PDV USA 2006 isolated from brain tissues were not present in isolates from lung, liver, or blood suggesting possible virus persistence in the central nervous system [44]. Furthermore, PDV USA 2006 has only a few amino acid substitutions in the P, M and F genes compared to the 1988 European Ulster/Netherlands strain of PDV [41]. Thus, the 2006 isolate from the United States might have emerged independently from 2002 PDV strains and multiple lineages of PDV might be circulating among endemically infected seals in the northwestern Atlantic [44].

#### 6.2.2. Eastern North Atlantic

**Figure 4:**
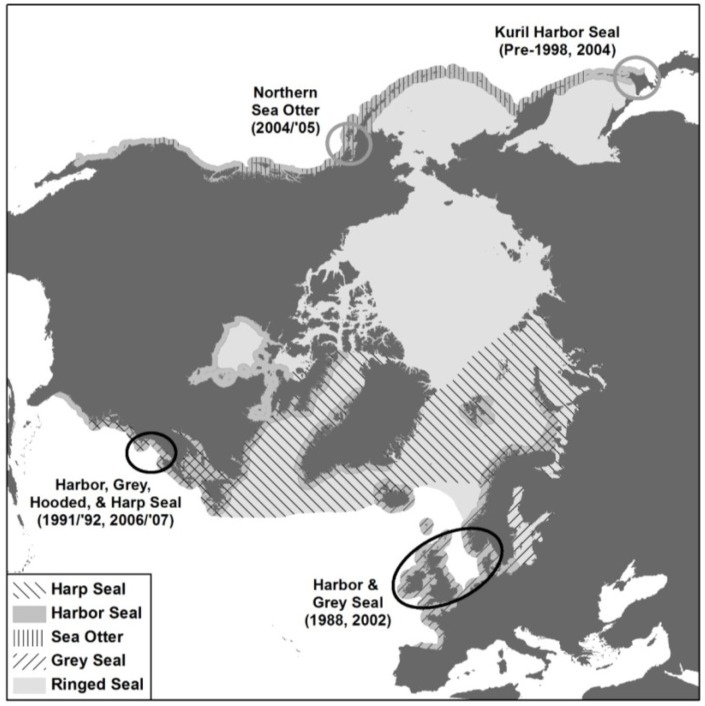
North polar azimuthal equidistant projection showing the location of 1988 and 2002 PDV epidemics in the northeastern Atlantic (lower right black oval); two mortality events in the northwestern Atlantic in which PDV infection was confirmed (left black oval); putative PDV-associated Kuril seal mortalities, Hokkaido, Japan (upper right grey circle); and presumptive PDV mortality in northern sea otters in southern Alaska (upper left grey circle). Distribution ranges or harbor, grey, harp, ringed seals and sea otters are shown.

The epidemiology of PDV in the northeastern Atlantic has been very well studied [41,199,200,201], particularly following the two European outbreaks (Figure 4) [94,202], and the volume of detailed data collected during both the 1988 and the 2002 events has been crucial in facilitating our understanding of the determinants and dynamics of the infection [116,158,203,204]. In particular it has enabled various mathematical models to be constructed from which predictions about the occurrence and impact of future events have been made [205,206,207,208]. The results of these different approaches have greatly enhanced our understanding of the modes and methods of disease transmission and the factors involved in determining the spread of the disease and the severity of the outbreaks. Most importantly detailed ecological and demographic data was collected from a very large proportion of beached carcasses or animals that died following admission into rehabilitation centers. This included the species, sex, age, condition and stranding location across all the European countries involved [174,201,202,203]. In addition, the occurrence of these two substantial outbreaks across largely the same geographical region, has allowed for a comparative approach [41,200]. Indeed some of the same researchers were involved in responding to and studying both epidemics which has increased the collective collaborative output as data were gathered using standardized protocols, similar techniques and approaches [202,209,210].

The initial cases of PDV in both outbreaks were identified in the Danish and Swedish Kattegat with the Danish island of Anholt being the breeding colony where the first cases were reported [3,116]. However, the reasons why this location is the starting point for the outbreaks remain elusive. An additional puzzling spatial feature was that the epidemics did not spread in a geographically coherent pattern. Whilst initially they spread linearly away from Anholt, new epicenters appeared as time progressed [41], suggesting vectors other than harbor seals (that generally forage coastally from haul-out sites) were responsible [41,200]. In the North Sea the grey seal, which was exposed but did not show overt infection or mortality [211] remains the most likely source. Indeed studies during the grey seal breeding season in the UK at the end of the 2002 epidemic found that blood samples from females and pups were PDV positive by PCR. Additionally, PCR positive pups with negative mothers were detected late in the breeding season suggesting transfer of virus across the colony [118]. However, whether grey seals are true reservoirs of infection or subclinical carriers is still to be determined [212].

The dynamics of infection differed regionally, particularly in Scotland where the prevalence of PDV antibodies was low following both outbreaks, more so in 2002 where the disease did not reach epidemic levels [200]. Lower mortality rates were also seen in England in 2002 compared to the European mainland and the earlier outbreak [202]. These region-specific patterns have therefore been important in improving our understanding of the factors regulating the severity of the outbreaks. Modeling the geographical spread of PDV and the utility of these models in the prediction of future events has been an active area of study, particularly since the 2002 outbreak. For example, spatially explicit modeling studies [213] used an individual-based model of seal movement, using tracking data obtained from harbor seals fitted with satellite relay data loggers, to combine realistic representations of animal behavior with traditional SEIR models [214]. It was concluded that the most important factors that affect the regional and temporal spread of infection are linked to the contact rate between infective and susceptible individuals, and that animal movement and haul-out connectivity are highly influential. Since life history stage will also affect activity as animals spend more time on land during breeding and molt [215], season is also a key factor. This approach could be expanded and enhanced in future to include the movements of grey seals as potential vectors or reservoirs of infection.

Further comparative modeling studies combining antibody prevalence serology data with a simple SEIR model found some interesting differences between the two outbreaks in the UK [202]. Overall the model suggested that there was a 27% (95% CI: 8% to 43%) fall in R_0_ the basic reproductive rate (*i.e.*, the number of cases generated by an infected individual during the infectious period in an uninfected population and is a combination of transmissibility, contact and duration of infectiousness) of the virus between outbreaks. However, viral transmission characteristics were similar throughout the UK and R_0_ was uniform within each epidemic. Thus the differences appear to have been mainly due to differences in case mortality. Seasonal differences in behavior resulting in more intense rather than more frequent contact between infectives and susceptibles may have resulted in differences in viral dose and the observed difference in case fatality rate. Higher mortality was seen in the fast growing European populations [216] where animals are not likely to be in poor condition, suggesting that other factors such as immunogenetic differences or immunotoxic effects of persistent organic pollutants may be at play [200].

The impact that an epidemic has on the future population dynamics of the species is determined not only by the overall mortality rate but also by the sex and age classes that are affected. Both epidemics had differential effects, particularly on the age classes that died [217]. Mortality among the young (<1 year) and older (>4 years) age classes was significantly higher than among the sub-adults, and males suffered significantly higher mortality than females. The study concluded that genetic susceptibility could not be the cause of this difference as adult mortality was higher than offspring mortality but that contact rate and susceptibility were likely to be strongly age and sex-specific. However, immunogenetic differences could explain the regional variation seen in mortality rates. For example, a study of MHC class I genes (which are critical for antigen presentation to T and NK cells), found presence/absence differences for some polymorphisms between UK harbor seal populations in southeast Scotland that were PDV survivors, and from southeast England that had died from the infection. However, the sample sizes were too small to draw firm conclusions [131]. This intriguing difference, and the variation identified by McCarthy *et al.* [65] for other immune-related genes, need to be investigated further. Nonetheless, life-long immunity to infection and the rate of acquired immunity development are key drivers of mortality patterns and spread of infection. The effect of acquired immunity on a simulated population of harbor seals subject to repeated PDV outbreaks was modeled and it was concluded that life-long immunity could actually impede the evolution of genetic disease resistance by maintaining susceptible genotypes in the population [205]. However, PDV mediated selection could still drive significant increases in resistance allele frequencies within a century, which would in turn buffer the impacts of future epizootics.

The population level impact and the consequences of repeated PDV epidemics for the long-term population persistence is an essential consideration in the conservation and management of the harbor seal in European waters [218]. This question was addressed using a stochastic model to analyze the long-term impacts on population dynamics [219]. However, in a response to this work, Lonergan and Harwood [220] indicated that their initial analyses did not include immunity in individuals that had survived previous outbreaks. This had a critical impact on the quasi-extinction risk estimates for harbor seals in Europe, where the estimated inter-epidemic interval for PDV is around 14 years [219]. Once included, the risk of a population decline to 10% of an initial population of 50,000 over 100 years was about 0.05 and if all the survivors of previous events are immune this declined to less than 0.01. These scenarios were for rapidly growing populations such as is currently seen in the Wadden Sea [221,222] and extinction risk is understandably more serious for populations that are declining due to other factors, such as those in some regions of Scotland [223]. Of some note is that European populations have largely recovered to their pre-2002 outbreak sizes, and although the recovery was delayed in some populations, particularly those in the Wash, SE England, they too are now increasing rapidly [224]. Consequently local population abundance, haul-out density, animal movement and the seasonal timing of any future outbreak could all affect contact rate. In addition, a recent study investigated the effect of maternal immunity on estimating the inter-epidemic interval for PDV in Europe [225]. Depending on the model assumptions, this could range from 6 to 12 years. Thus, risk predictions associated with repeated PDV outbreaks for declining populations remain challenging.

#### 6.2.3. North Pacific

The western Arctic, Bering and Chukchi Seas, and North Pacific Ocean have large and diverse populations of potentially PDV-susceptible marine mammals and some species such as ringed seals and bearded seals have a continuous distribution from the eastern Canadian Arctic to Alaska. Furthermore, some of these species including the northern sea otter, Steller sea lion (*Eumetopias jubatus*), northern fur seal (*Callorhinus ursinus*) and harbor seal, have experienced population declines through part of their range [180,226]. Although the cause of these declines is likely multifactorial, the role of infectious diseases requires investigation [227]. In Canada the most westerly PDV seropositive ringed seals were sampled at Paulatuk, NWT, close to the Alaskan border, in 1993 [12]. Early sero-surveillance in Alaskan waters using a CDV neutralization test, found no evidence of infection in ringed seals, harbor seals (*P. vitulia richardsi*) spotted seals (*Phoca largha*), bearded seals (*Erighnathus barbatus*), ribbon seals (*Histriophoca fasciata*), Steller sea lions or Pacific walrus (*O. rosmarus divergens*) sampled in the Bering Sea between 1984 and 1988 [6]. Later re-testing of the same sera found low or equivocal PDV plaque reducing antibody titers in some animals (Duignan and Nielsen unpublished).

Further south in Alaska, including the coasts of southeastern Alaska, Kodiak Island and Prince William Sound, harbor seals were sampled for serology between 1976 and 1999 [177]. While two of 160 seals had low PDV neutralizing titers, these were assumed to be false positive reactions [177]. A survey of 165 Steller sea lions in the same geographic region sampled between 1998 and 2000 found only one pup with neutralizing antibodies against dolphin and porpoise morbillivirus, but not PDV or CDV, and was negative by a competitive ELISA assay [180]. The authors concluded that this too was probably a false positive reaction. In Boundary Bay, British Columbia, a sample of 21 harbor seals (14 adult, 7 juvenile) were seronegative for all morbilliviruses [106]. Northern sea otters sampled in the western Aleutian Islands and at Elfin Cove in SE Alaska in 1997, and southern sea otters sampled in Monterey Bay, California, from 1995 to 2000 were all seronegative by ELISA [178].

The first compelling evidence for the presence of a morbillivirus in Pacific marine mammals was the discovery of 40% seropositivity in a PDV neutralizing test of live-captured sea otters from the Eastern Aleutian Islands sampled in 2004 and 2005 [117,181]. This stock of northern sea otters had declined dramatically from 74,000 to just under 9000 between the 1980s and 2000, and in 2006 large numbers of deaths were recorded in in south-central Alaska (Figure 3). However, although there was only equivocal evidence of morbillivirus pathology in examined animals, morbillivirus nucleic acid was amplified from 10% of nasal swabs of healthy live-captured otters and from tissues (lung, lymph node or brain) of three of nine beached carcasses found between 2005 and 2008 [117]. Partial sequence analysis of the P gene showed identity with PDV from Europe 2002 and close alignment with PDV from Europe 1988 and USA 2006 [44,117]. No sequence data was available for the more conserved and functionally significant H glycoprotein gene so speculation as to whether PDV arrived in Alaska along the Arctic Ocean coastline following the 1988 epidemic or one of the subsequent events is premature. More likely is the hypothesis that several lineages of PDV are circulating in endemically infected pinniped populations of the North Atlantic as occurs with CDV in terrestrial mammals [44,45]. If PDV has been present in the area since 2004, it is intriguing that there have been no confirmed PDV mortality events on the Alaskan coast. Northern sea otters are sympatric with PDV-naïve harbor seals in the Eastern Aleutian Islands and throughout the area of sea otter mortality in 2006. Further mortality surveillance of pinnipeds in this region is warranted.

Northern sea otters also occur along the Pacific coast as far south as northern California. In Washington State a mortality event occurred between July and August 2000 but the etiology was never established [182]. Retrospective serology on otters sampled from 1992 to 1997 were negative in a PDV and CDV competitive ELISA [179]. However, otters sampled later in 2001 were 80% (24/30) positive to CDV in a neutralization test while 10% (3/30) were positive in 2011 [182]. Differential serology suggested that CDV was the pathogen but PCR testing of nasal swabs proved negative. The conclusion was that terrestrial origin CDV was the cause and a precedent for this was the CDV epidemics among Baikal and Caspian seals in Asia [1,228]. Further south on the US Pacific coast, there has been no evidence for morbillivirus infection in pinnipeds or sea otters [179].

Marine mammal disease surveillance is more sporadic in the western North Pacific. A serologic survey was conducted on Kuril harbor seals (*P. vitulina stejnegeri*) at three locations on the southeastern coast of Hokkaido, Japan, between 1998 and 2005 [183]. Using an ELISA with PDV or CDV antigen, it was shown that at Nosappu, seals were PDV-seropositive in 1998 (50%), 2003 (5%), 2004 (1%) and 2005 (1%). A similar pattern was observed further south at Erimo where the prevalences were 13% (1999), 7% (2003), 50% (2004) and 0% (2005). Sixteen seals sampled at Akkeshi, between the previous two sites, were negative in 2004 and 2005. The authors discussed another report in Japanese [184] in which 19/23 (83%) of Kuril harbor seals from Hokkaido sampled in 1996 and 2/2 sampled in 1997 were PDV seropositive. Furthermore, greater than 50% of an unspecified number of Steller sea lions and spotted seals sampled between 1994 and 1998 tested positive. While no data are provided on stranding rates, mortalities or other diagnostic tests, the authors postulate that epidemics may have occurred around Hokkaido (Figure 3) prior to 1998 and again around 2004 [183]. Further research effort is certainly required to obtain a clearer understanding of morbillivirus epidemiology in this region.

#### 6.2.4. Southern Oceans

To date there is very little conclusive evidence for PDV in southern hemisphere pinnipeds. Antarctic crabeater (*Lobodon carcinophagus*) and leopard seals (*Hydrurga leptonyx*), but not Weddell seals (*Leptonychotes weddellii*) showed serological evidence of CDV exposure but there have been no confirmed mortalities [188]. Infection was likely introduced to Antarctica with expedition sled dogs. A serological survey of a small number of Weddell seals, Ross seals (*Ommatophoca rossii*, and crabeater seals from the pack-ice off Queen Maud Land, Antarctica sampled in 2001 showed no detectable PDV antibody levels [111]. In the New Zealand sub-Antarctic islands, a number of sea lions (*Phocarctos hookeri*) and New Zealand fur seals (*Arctocephalus forsteri*) had PDV neutralizing titers but there was no evidence of disease [185]. Titers in adult female New Zealand sea lions that died from a *Campylobacter spp*. septicemia epidemic in 1998 were too low to implicate PDV in the deaths and there were no morbillivirus lesions in tissues [186]. In Australia, a serological survey was conducted on 125 adult female Australian fur seals (*A. pusillus doriferus*) sampled at Kanowna Island rookery in Bass Strait between 2007 and 2009 [187]. A VeroDogSLAM plaque reduction assay for CDV was used but all animals were negative [187].

## 7. Conclusions and Future Directions

Since the discovery of PDV in 1988, it has become the most ecologically significant pathogen of pinnipeds, certainly in the northern hemisphere. At the First International Symposium on Morbillivirus Infections in Hannover, 1994, a workshop was convened to discuss the current understanding on the new marine mammal pathogens in the morbillivirus genus [15]. In retrospect, our knowledge at that time was quite limited. We understood the basic pathology and serology because of the similarities with CDV, could determine antigenic and genetic relationships to create rudimentary phylogenetic trees, and were beginning to grasp the intricacies of PDV epidemiology. Twenty years later, this review is testament to a huge corpus of research that has been generated largely because of the continued high profile of PDV as an agent of mass mortality for European harbor seals and smaller die-offs in eastern North America, and the recent emergence of infection in the North Pacific. The Princeton RAPIDD workshop was convened at this juncture to capture the advances in disparate fields of research pertinent to this deadly infection, to identify significant knowledge gaps, and to harness and channel the expertise of the assembled scientists into coordinated investigations on viral pathogenesis, immune response, phylogenetics, ecology and population dynamics.

Significant advances in understanding the pathogenesis of PDV have been made by the discovery of specific host cell receptors (CD150 and PVRL 4) that determine tissue (lymphoreticular cells, epithelial cells) and host specificity. Vero cells expressing canine CD150 receptor have since been developed to enhance our capacity to isolate wild-type PDV from field cases and as reagents for serology tests. However, we continue to speculate on the mode of entry of PDV into cells of the central nervous system. Does the virus persist in the protected environment of the brain and even acquire strain differences as has been suggested by some research findings? What determines host specificity and why are some species so susceptible to infection while others appear resistant to clinical disease? Could there be qualitative and quantitative differences in the distribution and affinity of relevant receptors? Answers to some of these questions may be provided by study of seals affected by field infection to assess the distribution of virus in tissues of different species infected by known viral strains. However, understanding of the molecular mechanisms will probably require development of *in vitro* models to assess attachment, infectivity and replication of the virus in specific cell types. Such studies will provide a greater insight on pathogenicity but will also facilitate the development of more sophisticated vaccines or therapeutics for endangered species such as the monk seals discussed in this review.

Considerable advances have been made in understanding the immune response of pinnipeds to PDV field infection and to vaccination by various attenuated and subunit vaccines as reviewed. Although we are closer to deployment of an inactivated vaccine as a strategy to protect the potentially vulnerable Hawaiian monk seal, further work is required to model the efficacy of such an approach. Better characterization of the immune response of different species of pinniped cells to different variants of PDV is required using *in vitro* approaches, and animal models to tease apart the genetic determinants, role of cytokines in immune response and immunosuppression (Th1 *versus* Th2 responses), and the components of cellular and humoral immunity.

Advances in molecular biology in the past 20 years have greatly enhanced our capacity to extract and sequence the morbillivirus genome reducing sequencing errors and the effects of adaptation to cell cultures. Complete sequences for the major genes are available for PDV from both European epidemics and from the USA 2006 mortality event affecting harbor seals. These will facilitate the resolution of the phylogenetic classification of PDV and its relationship with other members of the genus. However, similar sequence data are not available for other potential host species and are particularly lacking (apart from partial P gene sequence) for the harp seal. As this species is still the best candidate for reservoir of infection in the greater North Atlantic ecosystem, isolation of the virus and mapping the genome of PDV from harp seals should enable a better understanding of the spatial and temporal dynamics of infection. Similarly, more complete genetic mapping of the northern sea otter PDV genome is needed to determine the origin of this virus and its relationship to the North Atlantic variants.

The two major epidemics in Europe provided an unprecedented opportunity to develop various mathematical models from which predictions about the recurrence and impact of future events. In particular, the distribution of the outbreaks across largely the same geographical region, has allowed for a comparative approach with collaborative teams in several countries teasing apart the effects of time, space and seal ecology on disease transmission and expression. The use of individual-based models of seal movement from satellite tracked animals enabled researchers to combine realistic representations of animal behavior with traditional SEIR models and show how contact rate, animal movement, and haul-out connectivity are highly influential in epidemic dynamics. Significant knowledge gaps still exist for the northeast Atlantic and not least is where does PDV reside in the inter-epidemic interval? Does the more disease resistant grey seal play a role as vector around the coasts of Europe? What role does the eastern harp seal population play? What are the determinants of case fatality rate in harbor seals across metapopulations? How do immunogenetics and immunotoxicology impact the epidemiology? Developments in genomic technology now allow us to take a population genomic approach to assess genetic contributions to intra and interspecies differences in PDV susceptibility, building on the earlier studies of neutral markers and candidate genes.

In the western North Atlantic PDV appears to have been endemic in the larger and more diverse populations of pinnipeds for quite some time. While harbor and grey seals are found on both sides of the Atlantic, a key difference is the very large harp seal population that is seasonally resident on the sea ice of Maritime Canada and in close proximity to both grey and harbor seals. A second significant difference between east and west is that grey seals in UK waters, where the largest European population resides, breed in November, well before harbor seal breeding season in early summer. Therefore by the time the PDV epidemics started in Europe in April or May, the grey seal juveniles were already six months old and probably less vulnerable to infection. By contrast, in Canada and Maine, grey seal pups are born in February, harp and hooded seals in March, and harbor seals from April through early summer. Thus although there is high herd immunity in harp and gray seals, and moderate in hooded and harbor seals, there is an enormous influx of susceptible animals into the ecosystem each spring/summer. In this scenario, PDV has the potential to cause repeated but limited mortality events that can involve multiple species, mostly juveniles, but also older susceptibles for species like the harbor seal with lower herd immunity. While pinniped populations are higher in Atlantic Canada and the USA, large-scale epidemics as occur in Europe are never likely to occur because of the level of herd immunity. While this much we know, there is much more to understand about the dynamics of viral transmission and maintenance in this complex multi-host ecosystem.

Perhaps the greatest unknown to emerge from the RAPIDD workshop is the status of morbillivirus infection in the North Pacific. A PDV-like virus appears to have crept silently into central Alaska in the early 2000s and may or may not have been associated with a mortality event in northern sea otters in 2006. Preliminary data suggest that infection, based on serology and PCR, may now also be widespread in pinnipeds in the region. Given the propensity for PDV to cause epidemics in naïve phocid populations, the apparent lack of mortality among potentially vulnerable sympatric Pacific harbor seals is difficult to explain. Partial sequence data on the sea otter morbillivirus P gene indicates similarity to PDV from the North Atlantic; however, virus isolation and substantially more sequence data are required before the phylogenetic relationships can be determined. Equally intriguing is whether or not this virus or yet another variant is circulating in the coastal waters of Japan and Russia and possibly causing localized epidemics among Kuril harbor seals. Further surveillance in the North Pacific in general is required before we can speculate on the ecology of PDV in this region.
